# Supplementary material for: Identification of Neural and Non-Neural Origins of Joint Hyper-Resistance Based on a Novel Neuromechanical Model
Source: IEEE Trans Neural Syst Rehabil Eng. Author manuscript; Available in PMC 2024 Apr 21. (PMC11032725; doi:10.1109/TNSRE.2024.3381739)
Supplement: supp1-3381739 [file NIHMS1982336-supplement-supp1-3381739.docx]

**Supplementary material**

**S1. Initial guess**

As our non-linear optimization problems had many local optima, we solved each optimization problem using 10 initial guesses and selected the solution that resulted in the lowest cost function.

Table S1: Different initial guesses that are used to solve the optimization problem.

|  | IG 1 | IG 2 | IG 3 | IG 4 | IG 5 | IG 6 | IG 7 | IG 8 | IG 9 | IG 10 |
| --- | --- | --- | --- | --- | --- | --- | --- | --- | --- | --- |
| a_ext_ | 0.0100 | 0.0050 | 0.0010 | 0.0001 | 0.0100 | 0.0500 | 0.0100 | 0.0100 | 0.1000 | 0.0300 |
| a_flex_ | 0.0100 | 0.0010 | 0.0005 | 0.0005 | 0.0050 | 0.0100 | 0.0010 | 0.0010 | 0.0500 | 0.0500 |
| k_R_ | 0.0100 | 0.0010 | 0.0020 | 1.0000 | 1.0000 | 3.0000 | 1.0000 | 5.0000 | 0.1000 | 0.5000 |
| k_Fpe_ | 0.1000 | 0.1500 | 0.2000 | 0.2000 | 0.2000 | 0.1500 | 0.1000 | 0.1500 | 0.2000 | 0.2000 |
| B | 0.1000 | 0.0500 | 0.0600 | 0.0300 | 0.0600 | 0.0200 | 0.0500 | 0.0200 | 0.0300 | 0.0300 |
| dt1 | 0.0050 | 0.0100 | 0.0030 | 0.0020 | 0.0050 | 0.0010 | 0.0020 | 0.0020 | 0.0010 | 0.0010 |
| x | Experimental joint kinematics | | | | | | | | | |
| **ẋ** | Experimental angular velocities | | | | | | | | | |

a_ext_ = baseline activation extensor; a_flex =_ baseline activation flexor; kR = reflex gain; kFpe = shift in passive force-length curve; B = damping coefficient; dt 1 = optimized mesh intervals from phase 1; x = state trajectories for joint angle; $\dot{x}$ state trajectories for angular velocities; IG = initial guess.

For most of the trials, different initial guesses only lead to small differences in kinematic outcome (i.e., pendulum trajectory) and optimized parameters (e.g., figure S1, panel b). However, for some trials that are sensitive to local optima, different initial guesses led to very different outcomes (e.g. figure S1, panel d). We analyzed which initial guesses led to the most optimal solution (smallest cost) , and found that almost all initial guesses led to the most optimal solution at least once underlying the importance to use an extended set of initial guesses (figure S1, panel a). In addition, when kinematic trajectories obtained based on the different initial guesses were similar, the underlying parameters were typically similar as well (figure S1, panel b). In panel b, we present two trials that had the largest range in variables when kinematic trajectories were similar based on different initial guesses.


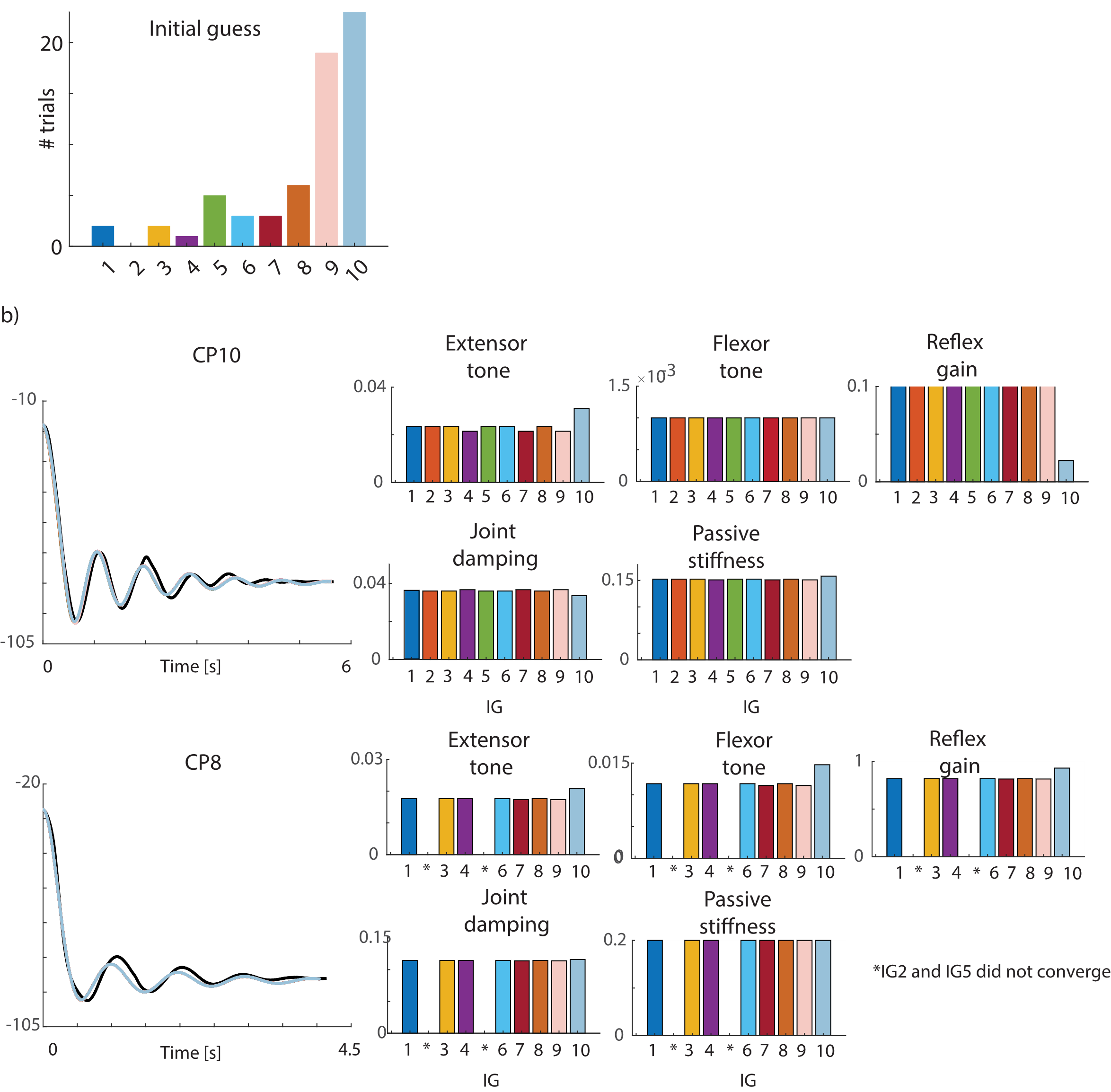


Figure S1: Analysis of initial guesses used. a) Different initial guesses used across all trials. Except for initial guess 2, all initial guesses are used to detect the best fit. b) Two trials representing the largest variability in parameters when kinematic trajectories were similar based on different initial guesses. Each color represents one IG.

**S2. Threshold calculation**

We divided average peak EMG response into two categories: (1) low EMG response when peak EMG < 0.01, and (2) high EMG response when peak EMG > 0.01. We defined the threshold based on visual inspection of experimental peak EMG data. All typically developing children were grouped in the low EMG response group, suggesting that our threshold makes sense.


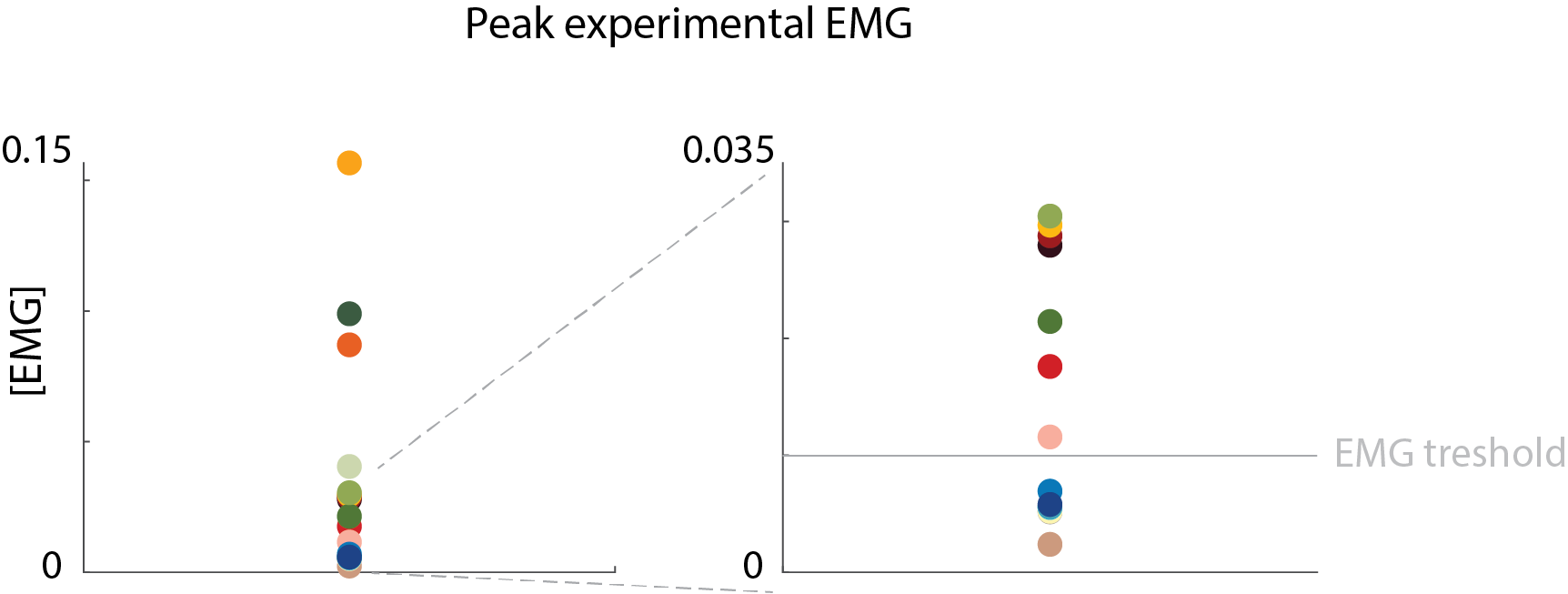


Figure S2: Threshold calculation. Average peak EMG for each subject is represented with one dot (left). Right figure is a close-up from the right figure.

**S3. Pendulum simulations for all participants and all trials**


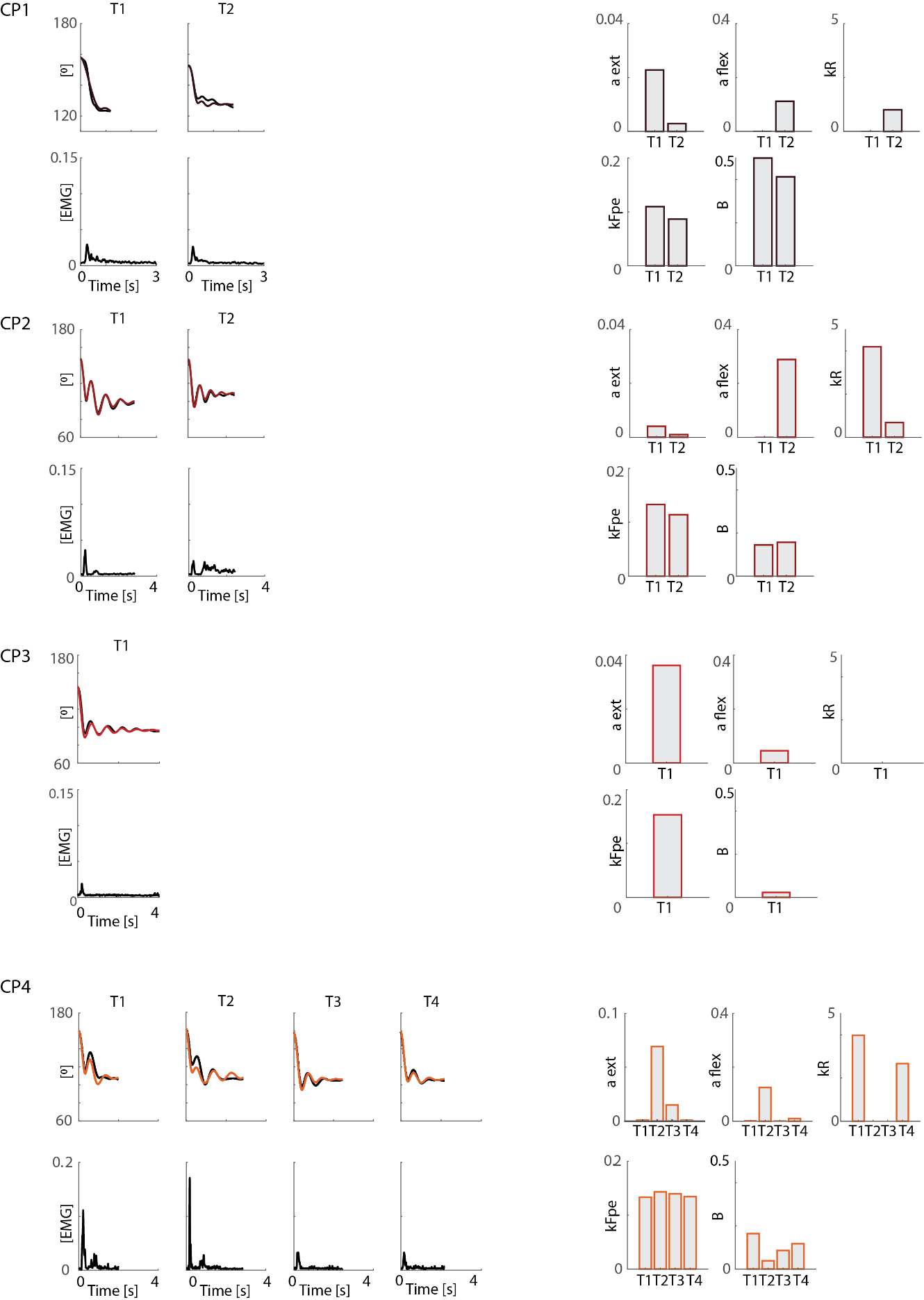


Figure S3: Experimental pendulum and EMG trajectories (black), simulated pendulum trajectories (color) and simulated parameters (right). A ext = baseline muscle tone for the extensor; a flex = baseline muscle tone for the flexor; kR = reflex gain; kFpe = shift in passive length-force curve; B = damping. (Part 1/5)


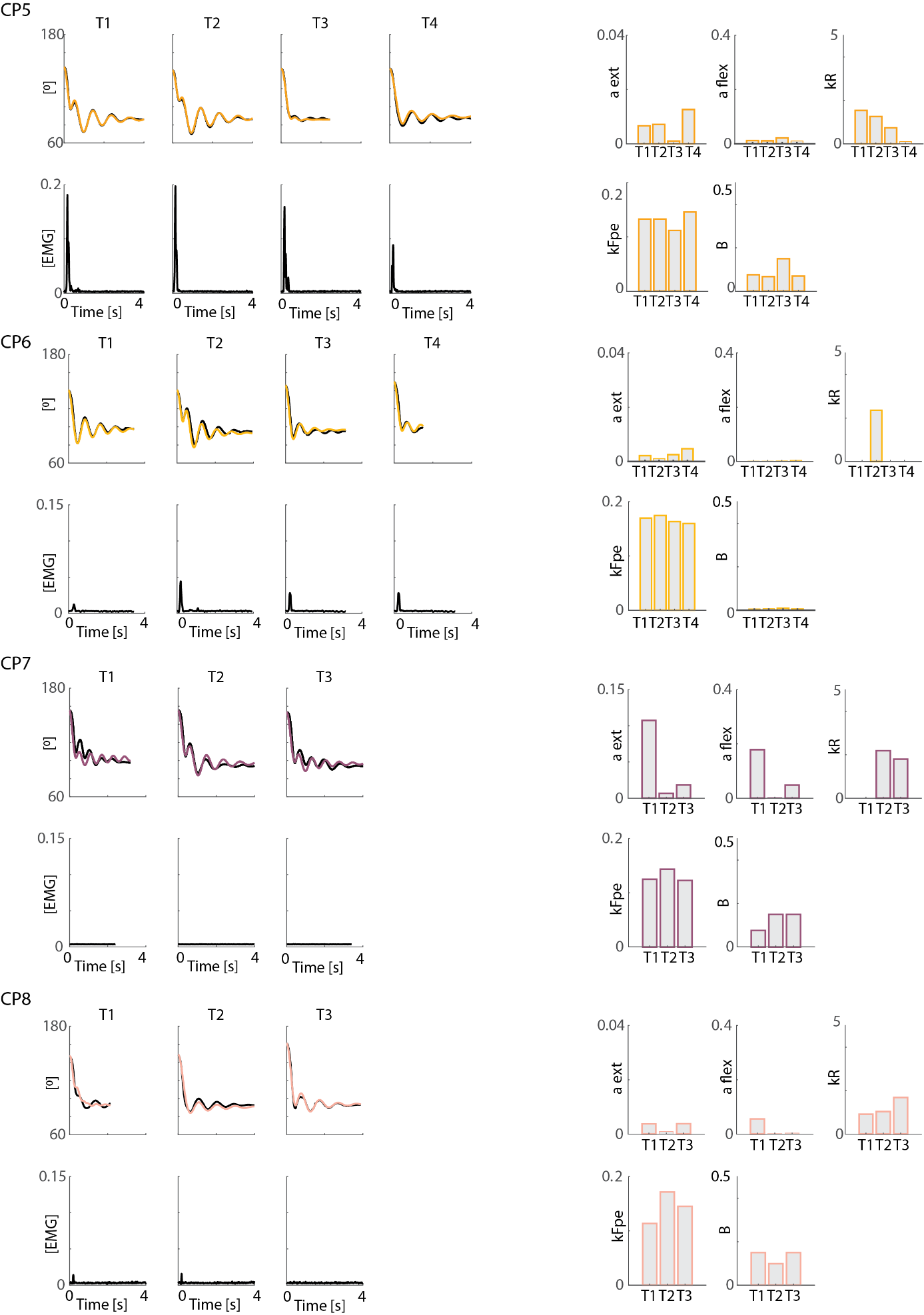


Figure S3: Experimental pendulum and EMG trajectories (black), simulated pendulum trajectories (color) and simulated parameters (right). A ext = baseline muscle tone for the extensor; a flex = baseline muscle tone for the flexor; kR = reflex gain; kFpe = shift in passive length-force curve; B = damping. (Part 2/5)


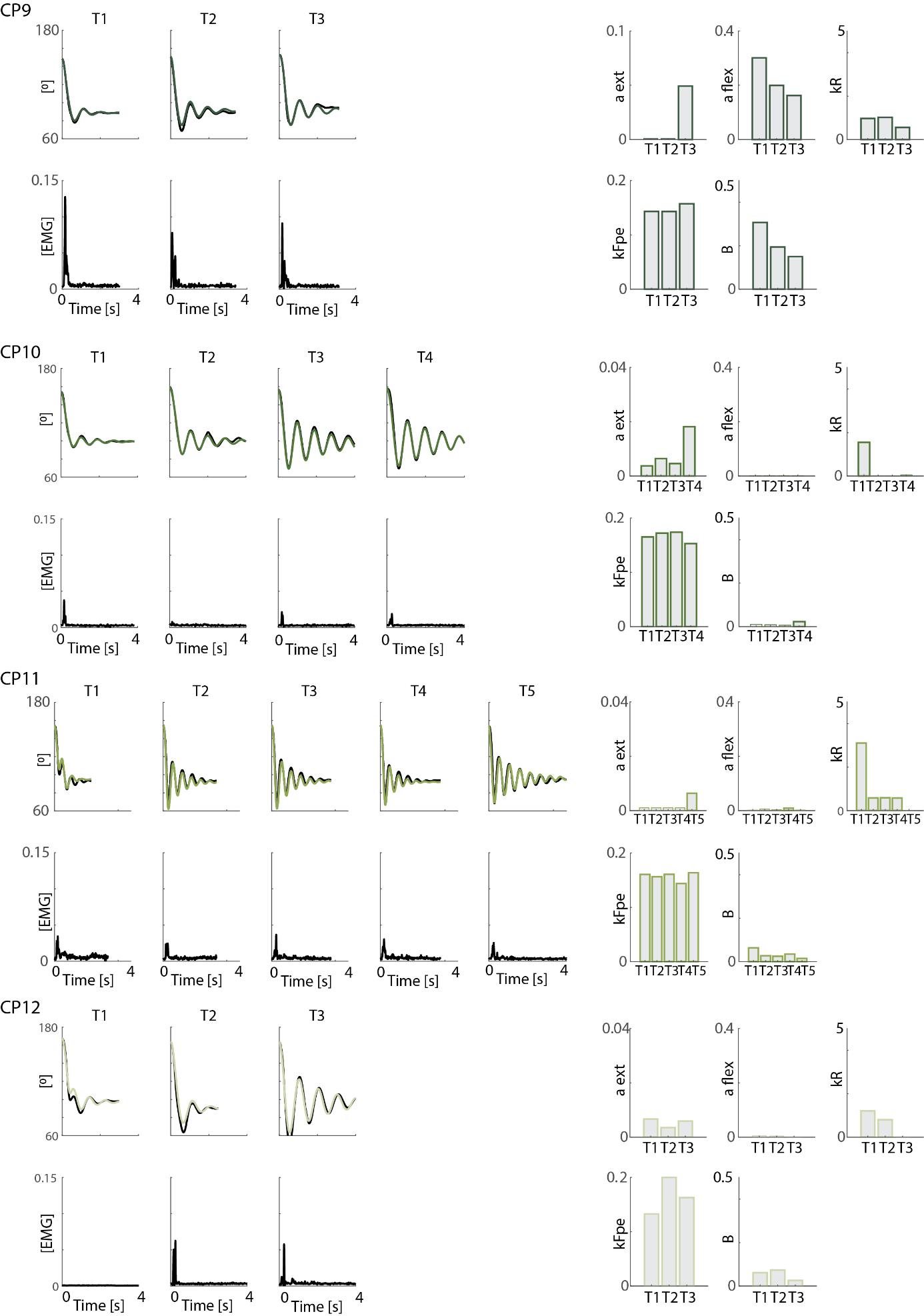


Figure S3: Experimental pendulum and EMG trajectories (black), simulated pendulum trajectories (color) and simulated parameters (right). A ext = baseline muscle tone for the extensor; a flex = baseline muscle tone for the flexor; kR = reflex gain; kFpe = shift in passive length-force curve; B = damping. (Part 3/5)


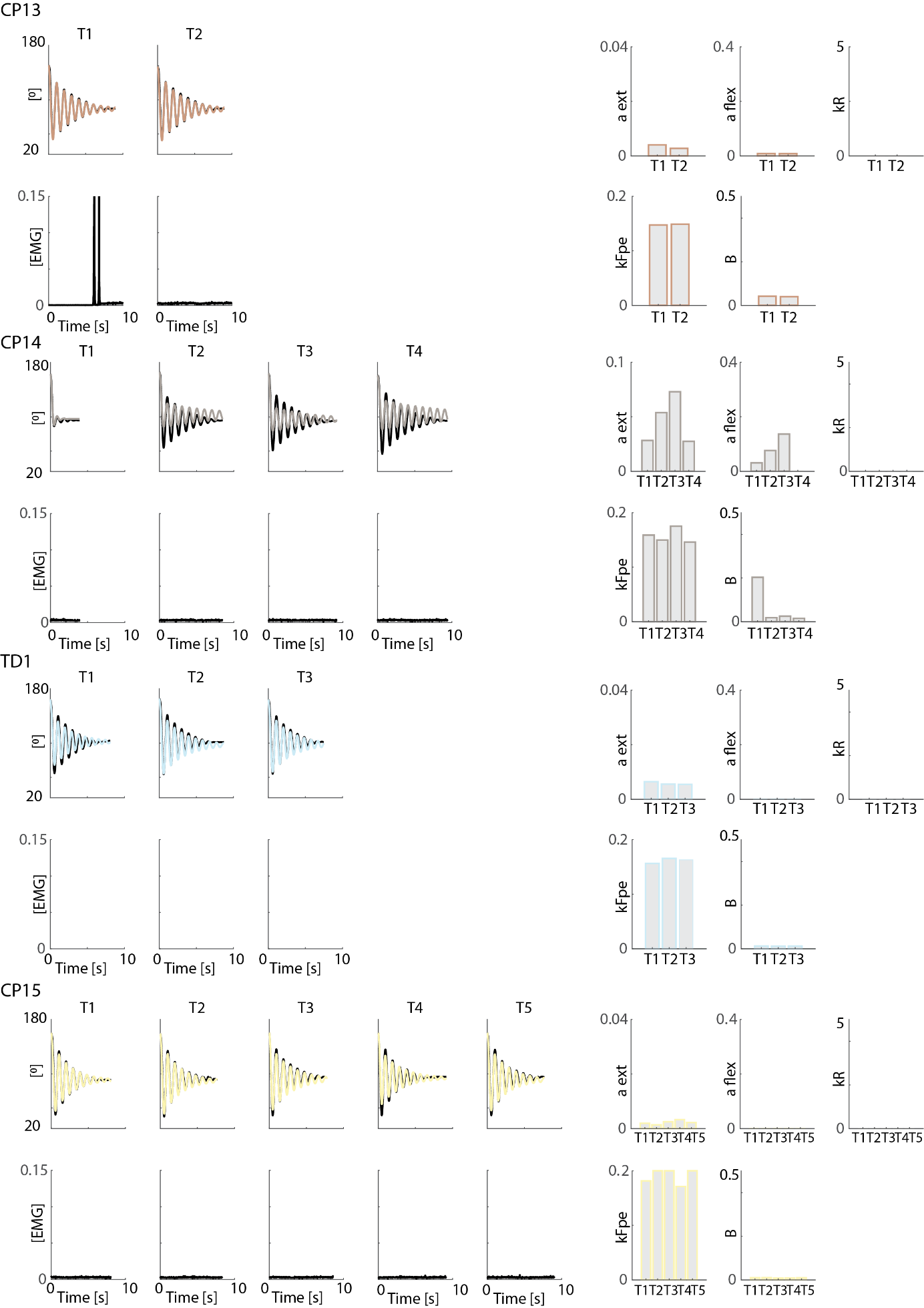


Figure S3: Experimental pendulum and EMG trajectories (black), simulated pendulum trajectories (color) and simulated parameters (right). A ext = baseline muscle tone for the extensor; a flex = baseline muscle tone for the flexor; kR = reflex gain; kFpe = shift in passive length-force curve; B = damping. (Part 4/5)


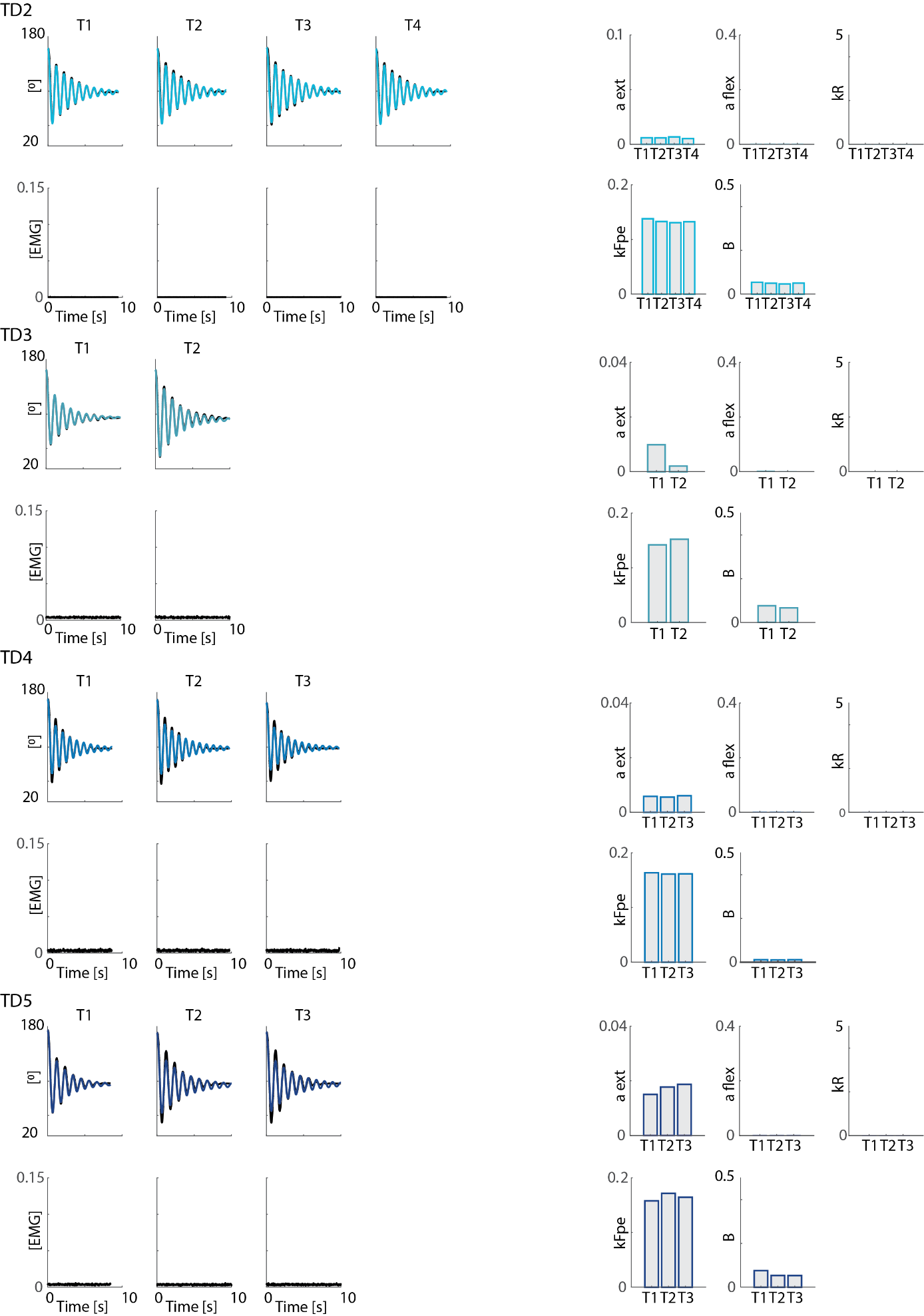


Figure S3: Experimental pendulum and EMG trajectories (black), simulated pendulum trajectories (color) and simulated parameters (right). A ext = baseline muscle tone for the extensor; a flex = baseline muscle tone for the flexor; kR = reflex gain; kFpe = shift in passive length-force curve; B = damping. (Part 5/5)

**S4. Sensitivity analysis for four exemplar trajectories**

We performed a sensitivity analysis for the representative children for whom results are shown in figure 3 (manuscript). These children were selected to span the range of kinematic trajectories observed in the experimental data. We modified each optimized parameter with +5% and -5% of the range (defined as max – min value) observed in the respective parameters across all trials of all children. In cases were reducing a parameter with -5% would cause us to violate to the lower bound (e.g., negative activations or reflex gains), we limited the reduction to this lower bound.

**
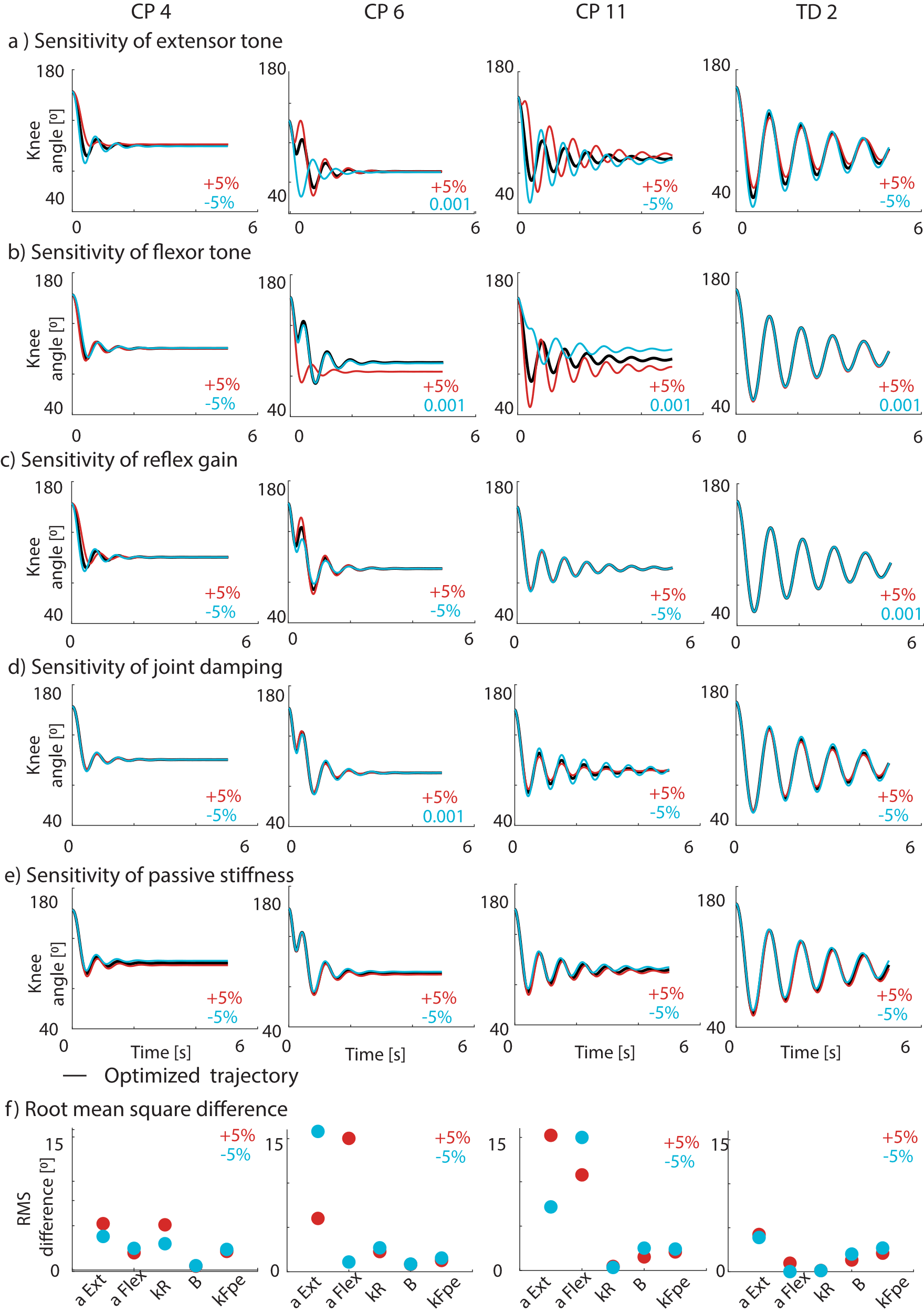
**

Figure S4: Sensitivity of four example simulated knee angle trajectories to the parameters. Parameters that optimized the fit between simulated and experimental data were individually altered by 5% of the range across all subjects and trials. a) Influence of extensor muscle tone. b) Influence of flexor muscle tone. c) Influence of reflex gain. d) Influence of joint damping. e) Influence of passive stiffness. f) Root mean square difference between optimized trajectories (black) and trajectories with altered parameters (red and blue). Red trajectories were obtained by increasing the parameter with 5% of the range. Blue trajectories were obtained by decreasing the parameter with 5% of the range.

**S5. Associations between kinematic features and simulated parameters.**


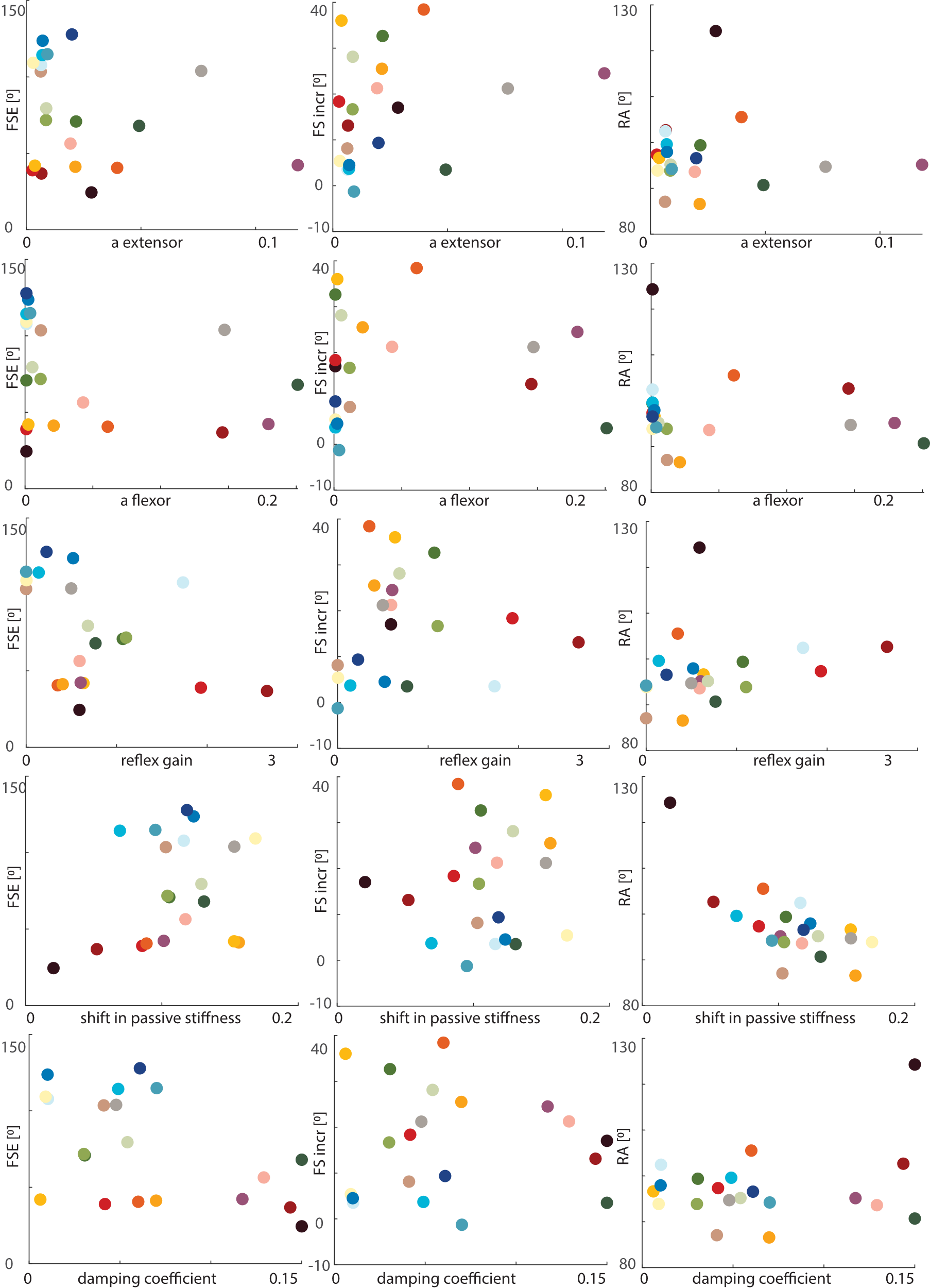


Figure S5: Associations between experimental kinematic features (y-axis) and simulated (x-axis) parameters. Every dot represents the average across all trials for one child. Typically developing children in blue. FSE = first swing excursion; FS incr = increase in first swing excursion after pre-movements; RA = resting angle.

**S6. Exemplar trajectories for the predicted effect of pre-movement.**


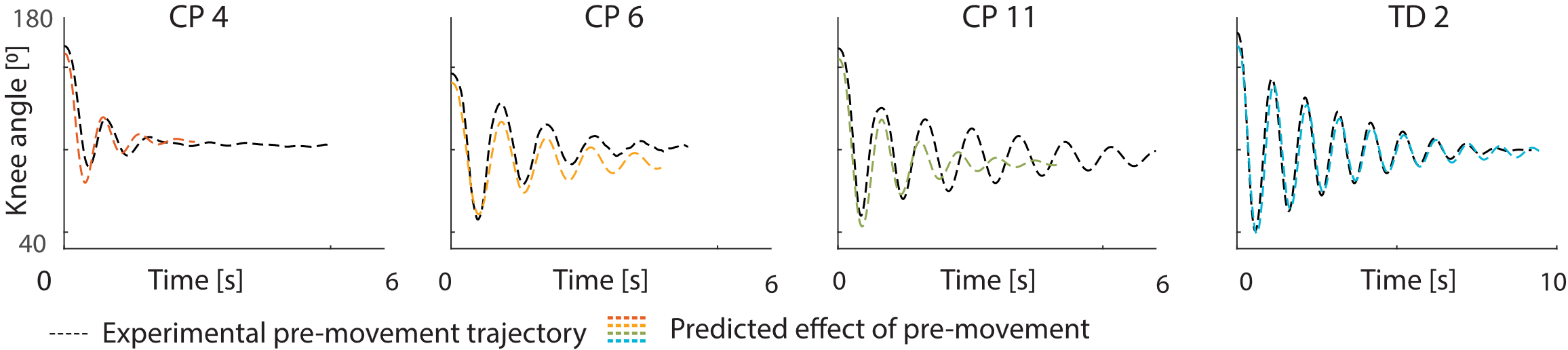


Figure S6: Exemplar trajectories for experimental and predicted effect of pre-movement. Black dotted lines are experimental pre-movement trajectories. Colored dotted lines are predicted pre-movement trajectories. CP = cerebral palsy; TD = typically developing.
